# Crystalline Characteristics and Their Influence in the Mechanical Performance in Poly(ε-Caprolactone) / High Density Polyethylene Blends

**DOI:** 10.3390/polym11111874

**Published:** 2019-11-13

**Authors:** Enrique Blázquez-Blázquez, Ernesto Pérez, Vicente Lorenzo, María L. Cerrada

**Affiliations:** 1 Instituto de Ciencia y Tecnología de Polímeros (ICTP-CSIC), Juan de la Cierva 3, 28006 Madrid, Spain; enrique.blazquez@ictp.csic.es (E.B.-B.); ernestop@ictp.csic.es (E.P.); 2 Grupo de Investigación “POLímeros: Caracterización y Aplicaciones”, E.T.S.I. Industriales, Universidad Politécnica de Madrid, José Gutiérrez Abascal 2, 28006 Madrid, Spain; vlorenzo@etsii.upm.es

**Keywords:** blend, polycaprolactone, polyethylene, crystallization, compatibility

## Abstract

Blends of poly(ε-caprolactone) (PCL) and high-density polyethylene (HDPE) have been prepared at different compositions in order to assess the effect of HDPE on gas transport and mechanical behaviors of PCL. Previous to this evaluation, a complete morphological, structural, and thermal characterization were performed using techniques, including SEM, contact angle, FTIR, differential scanning calorimetry, and X-ray diffraction with synchrotron radiation at small and wide angles. Low HDPE incorporations allow interactions to be established at interfaces in the amorphous regions and the enhancement of the mechanical performance. Consequently, the addition of a small amount of HDPE (ranging from 5 to 10 wt%) appears to be appropriate in certain bio-applications where a higher mechanical behavior is required.

## 1. Introduction

Poly(ε-caprolactone) (PCL) was first synthesized in the early 1930s by the Carothers group [1], and consists of a linear aliphatic polyester macrochains, exhibiting crystalline characteristics. It was soon commercialized given that one of the synthetic polymers has the ability to be degraded by microorganisms [2]. During 1970s and 1980s, attention was significantly focused on PCL and its copolymers for their use in drug-delivery devices. Nevertheless, it was overwhelmed by other resorbable polymers, like polylactides and polyglycolides. The birth of the field of tissue engineering involved the renaissance of interest in the PCL and its back into the biomaterials arena [3], since PCL exhibits superior properties (namely viscoelastic and rheological ones) when compared with other resorbable-polymer competitors [4,5,6,7,8]. PCL has additionally become a highly desirable candidate for applications like the controlled release of contraceptives in matrix implants [9,10,11], since a second surgery for the retrieval of the device could be avoided. This role is mainly associated with its biocompatibility and the FDA approval together with its slow biodegradation. Nevertheless, its structural bio-applications can be limited by its relatively low glass transition temperature, T_g_ (around −60 °C), and its low melting point, T_m_ (about 60 °C). These are the features that control its mechanical performance (which are particularly deficient under load bearing conditions, requiring considerable mechanical reinforcement) and its barrier characteristics (to oxygen, water vapor and other gases). The use of advanced fabrication technologies, like three-dimensional (3D) printing or electrospinning [12,13], and its blending with other polymers [14,15,16,17,18,19] or fillers [20,21,22,23], can promote the enhancement of those properties.

Non-degradable polymers, as well named as biointegrable or biostable, have long been used for treating a wide range of health problems. Polyethylene, poly(methyl methacrylate), polyurethanes, poly(tetrafluoroethylene), and silicone are some of the polymers commonly employed for applications, such as drug delivery systems, orthopedics, vascular grafts, tissue engineering, dentistry, and ocular applications. Polyethylene (PE) is one of those biostable polymers occupying a prominent position in medical applications. Different polyethylenes can be obtained: Low density polyethylene (LDPE), linear low-density polyethylene (LLDPE), high density polyethylene (HDPE), ultra high molecular weight polyethylene (UHMWPE), and cross-linked polyethylene (XLPE). Among them, HDPE and UHMWPE have widespread medical applications. The former has already been used in tubing for drains, catheters, and stents for distal malignant biliary obstruction [24,25], due to its outstanding toughness and resistance to fats. Rather significant amounts of some particulate fillers can be incorporated into HDPE by melt processing using standard methods. Moreover, it is a linear polymer with important advantages when using certain complex processing technologies. For instance, its extrusion under hydrostatic conditions leads to the alignment of the polymeric chains in the direction of extrusion and the effect is a high strength and highly modulus polymer material. If HDPE is synthesized with exceptionally high molecular weights, exceeding a million g/mol, UHMWPE is achieved. Its niche has been in hip prostheses [26,27,28,29,30] for over thirty years now.

Blends of biodegradable PCL and non-biodegradable HDPE might lead to improvements in deficient gas transport properties and mechanical responses exhibited by neat PCL. Why could HDPE be a good choice? There are not many investigations dealing with blends based on these two polymers but the incorporation of HDPE could be interesting because it is a biomaterial that develops a relatively high crystallinity, and its chemical structure is fairly analogous to that present in PCL. It crystallizes at temperatures higher than PCL as it is able to exert a positive effect on the crystalline PCL characteristics. Some articles in the literature also support this selection: considerable “mechanical” compatibility was observed [31] at the extremes of composition in LDPE/PCL blends; maintenance of PCL biodegradability [32] in blends containing up to a 30 wt% of HDPE was described, although the rate was reduced. Furthermore, the formation of ordered, unusually thick, lamellae PCL in contact with a PE substrate [33] was reported. Thus, the aim of the present work consists of preparing blends based on PCL and HDPE in the whole composition interval, as well as the evaluation of their crystalline characteristics and their ultimate performance. Attention has been mainly focused on the low contents of HDPE to understand whether gas transport behavior and mechanical response of the PCL, which is the polymer of interest in this research, can be conveniently tuned. This blending methodology would constitute a good approach at low production costs. PCL biodegradability is expected to be somehow altered, but the influence might be acceptable at those low HDPE contents, mainly in medical applications that involve slow degradation rates, for instance, the manufacture of long-term drug delivery devices, as well as contraceptives in matrix implants for women (this latest point is out of the scope of this article). All the characteristics found when blend composition is changed will be discussed in terms of modifications at the interfaces and in the crystalline regions of both components, as well as the influence of these variations in the final properties in the blends. Numerous techniques have been required, including: scanning electronic microscopy (SEM); contact angles measurements; infrared spectroscopy with Fourier Transform (FTIR); differential scanning calorimetry (DSC); X-ray diffraction with synchrotron radiation at either small (SAXS) or wide angle (WAXS); experiments for gas transport behavior; uniaxial tensile stress-strain measurements, and; dynamic-mechanical thermal analysis (DMTA).

## 2. Materials and Methods

### 2.1. Materials

A commercially available polycaprolactone (purchased from Sigma-Aldrich, San Luis (MO), US) with an average molecular weight (*M_n_*) of 80,000 g/mol and a density of 1.1450 g/cm^3^, together with a metallocenic catalyzed high density polyethylene [34] (supplied by Repsol, Madrid, Spain) with an average *M_n_* of 93,700 g/mol and a density of 0.9499 g/cm^3^, have been used for obtaining blends with different compositions. 

### 2.2. Blends and Films Preparation

The contents in PCL were: 95%, 90%, 75%, 50%, and 25% in weight, and labeled as PCL95, PCL90, PCL75, PCL50, PCL25, respectively, and the corresponding homopolymers, PCL and HDPE were named as PCL100 and PCL0. They were prepared at 170 °C and at 100 rpm for 15 min in a Haake Minilab twin-screw extruder (Thermo Electron Corporation, Waltham (MA) US) with a volumetric capacity of 7 cm^3^ using co-rotating conical screws.

After blending of the two components and homogenization of the mixture, films of the different materials were obtained by compression molding. A Collin press (Ebersberg, Germany) was used for that purpose, between hot plates (160 °C) during 5 min and applying a pressure of 2.5 MPa. Subsequently, fast cooling was applied from 160 °C down to ambient temperature between steel plates refrigerated with water (average cooling rate around 80 °C/min). The thickness of these films ranged from 0.15 to 0.20 mm.

### 2.3. Characterization of the Samples

#### 2.3.1. Scanning Electron Microscopy

These micrographs were acquired at room temperature in an Environmental Scanning Electron Microscope PHILIPS XL30 ESEM (Leuven, Belgium)working at 25 kV, using a secondary electron (SE) detector and a magnification of ×2000 for the morphology studies. The samples were cryofractured prior observation.

#### 2.3.2. Contact Angle

An optical tensiometer (Attension Theta, Stockholm, Sweden) was employed for measuring the apparent contact angles, by means of the sessile drop method and applying a conventional analysis of the drop shape. For that method, Milli-Q grade water was used. The values of *θ_w_* were taken as the initial water contact angle in static conditions by using a drop of 5 μL volume. At least five measurements were performed in different regions of the sample, and the corresponding averages are the values reported for *θ_w_*.

#### 2.3.3. Fourier-Transform Infrared Spectroscopy

FTIR-ATR spectra of the two homopolymers and the several blends were acquired with a Perkin Elmer spectrometer coupled to a device of attenuated total reflectance (ATR, Waltham (MA), US). Four consecutive scans with a resolution of 1 cm^−1^ were recorded for each sample, performed in a spectral range of 600−4000 cm^−1^. The absorbance of the spectra was normalized to the intensity of the peak at 2722 cm^−1^.

#### 2.3.4. X-Ray Diffraction

Real-time X-ray diffraction experiments were acquired in a synchrotron source (former beamline A2 of DESY-HASYLAB, Hamburg, Germany). The wavelength of the source was 0.150 nm, with heating experiments at a rate of 8 °C/min, in the temperature interval from 20 to 144 °C. The selected frame time was 15 s, meaning that the temperature difference between frames is 2 °C. Both WAXS and SAXS data were obtained by using a MARCCD detector, located at either 135, or 3000 mm from sample, respectively. The calibration of the spacings was carried out by means of the different orders of the long spacing (*L* = 65 nm) of a rat-tail cornea for the SAXS detector, and the several diffraction peaks of a semicrystalline poly(ethylene terephthalate) film for the WAXS detector. The A2tool program, implemented for data processing in the beamline, was used for converting the initial 2D images into 1D diffractograms, which were normalized in relation to the intensity of the primary beam. In addition, the background that came from the temperature controller, without a sample, was subtracted from those profiles.

#### 2.3.5. Differential scanning calorimetry

DSC experiments were performed in a TA2000 calorimeter (from TA Instruments, New Castle (DE), US) at a rate 20 °C/min (under dry nitrogen, and provided with a cooling device) in the temperature range from −80 to 200 °C. Sample weights of around 5 mg were used. The calorimeter was calibrated for temperature and enthalpy with different standards. For crystallinity determinations, a value of 135 J/g was considered for the melting enthalpy of the 100% crystalline PCL [35,36] while 290 J/g is that taken for HDPE [37].

#### 2.3.6. Gas Transport Behavior

Non-commercial equipment, which was built in our laboratory, has been used for measuring the permeability of the samples. The main part of the equipment is a permeation cell made of a stainless-steel filter holder of 47 mm (Millipore XX4404700), with 13.8 cm^2^ of effective area, and with two chambers separated by the membrane. In addition, a system for measuring pressure and a thermostatic bath for temperature control are provided. Moreover, sensors of both temperature and absolute pressure (MKS Baratron), were used for automated data collection. Diffusion experiments through the initially purged membrane were carried out for determining the gas permeability of the samples. In this respect, the low-pressure and high-pressure chambers separated by the film were both subjected to a high vacuum (around 10^−4^ mbar). A step variation of the pressure (after purging the system) was imposed on the high-pressure side of the membrane (*p_H_* = *p_0_* for time t > 0), while monitoring the pressure on the low-pressure side, *p_L_*. The leak curve was determined before each experiment by measuring the pressure variation with time in the low-pressure chamber in vacuum. Between runs, all membranes were degassed overnight under vacuum. Experimental determinations were performed at 30 °C and 1 bar of pressure with different gasses: Nitrogen, oxygen, and carbon dioxide. A relatively high reproducibility of the measurements was obtained, since the standard deviation for the different transport magnitudes is smaller than 5% of the mean values.

#### 2.3.7. Stress–Strain Response

The mechanical behavior of the samples has been determined from stress-strain measurements for PCL, HDPE, and the distinct blends. A dumbbell die was used for obtaining suitable shaped specimens from the initial sheets. The gauge dimensions of these specimens were 1.9 mm in width and 15 mm in length, while a range between around 0.15 and 0.20 mm was obtained for their thicknesses. An Instron Universal testing machine (calibrated with standard protocols) was employed for the tensile experiments, performed at room temperature and with 10 mm/min of nominal crosshead speed using a cell load of 100 N. The various mechanical magnitudes (Young´s modulus, yield stress and the area under stress-strain curve related to toughness) were calculated [38] from the distinct experiments. For each material, at least four specimens were analyzed and the reported results are the corresponding mean values. The error in the mean values is less than 10%.

#### 2.3.8. Dynamic Mechanical Thermal Analysis

A dynamic mechanical thermal analyzer (Polymer Laboratories MK II) was used for determining the viscoelastic properties. The tensile mode was selected, and for each sample the real (E′) and imaginary (E″) components of the complex modulus and the loss tangent (tan δ) were calculated at 1, 3, 10, and 30 Hz, in the temperature interval from −150 °C to 150 °C, using 1.5 °C/min as the heating rate.

### 2.4. Statistical Analysis

The experimental data for contact angle or mechanical parameters were statistically analyzed. One-way analysis of variance (ANOVA) was carried out and significant differences among blends were recorded at a 95% confidence level. Tukey HSD tests have been used for the multi-comparisons of the mean values for the different magnitudes in the diverse blends and homopolymers studied. The samples have been grouped and labeled according to the results of the post-hoc tests in their corresponding Figures (see below).

## 3. Results and Discussion

### 3.1. Characterization of Morphology and Interactions

**Figure 1:**
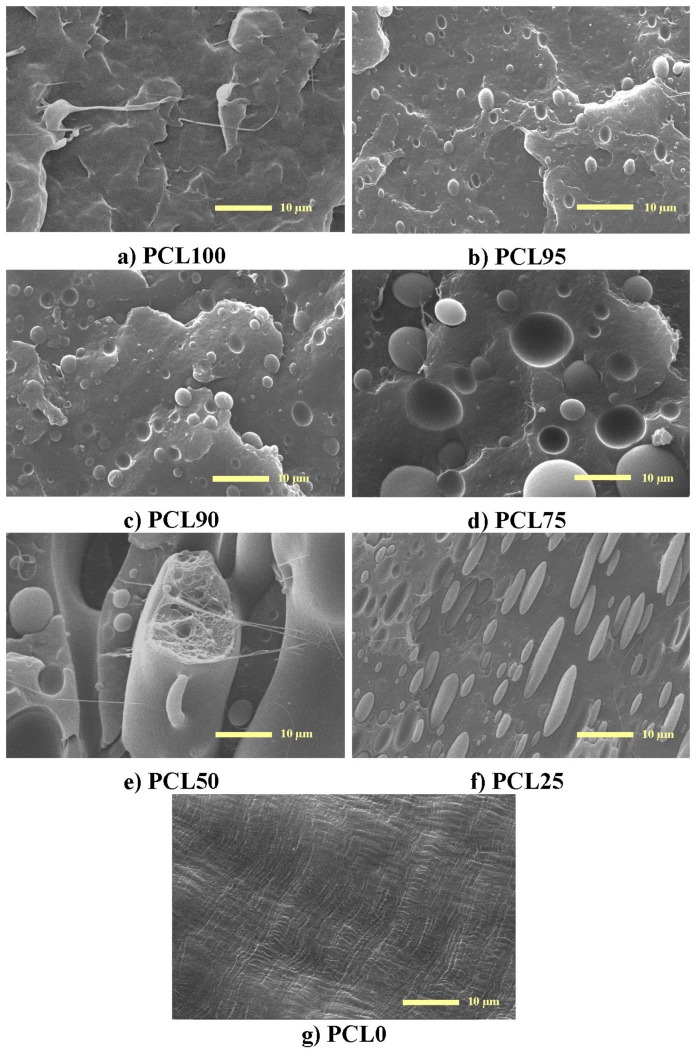
SEM micrographs of longitudinal fracture surface for: **a**) poly(ε-caprolactone) (PCL); the different blends (**b**) PCL95, **c**) PCL90, **d**) PCL75, **e**) PCL50, **f**) PCL25); and **g**) high-density polyethylene (HDPE). The scale bar is referred to 10 µm and magnifications are for all of them x 2000.

Figure 1 shows the micrographs taken from the fractured surface at different contents by using scanning electron microscopy (SEM). When PCL is the major component (samples PCL95, PCL90, and PCL75, micrographs b), c) and d), respectively), uniformly distributed spherical domains of HDPE can be observed all over the PCL matrix. The size of these dispersed domains increases as the content of the minor HDPE component is raised. The presence of these droplets reveals the existence of phase separation that can be explained in terms of the existing difference between the Hildebrand solubility parameters of both polymers (16.7 and 19.9 (MPa)^1/2^ for PE and PCL, respectively) [39].

**Figure 2:**
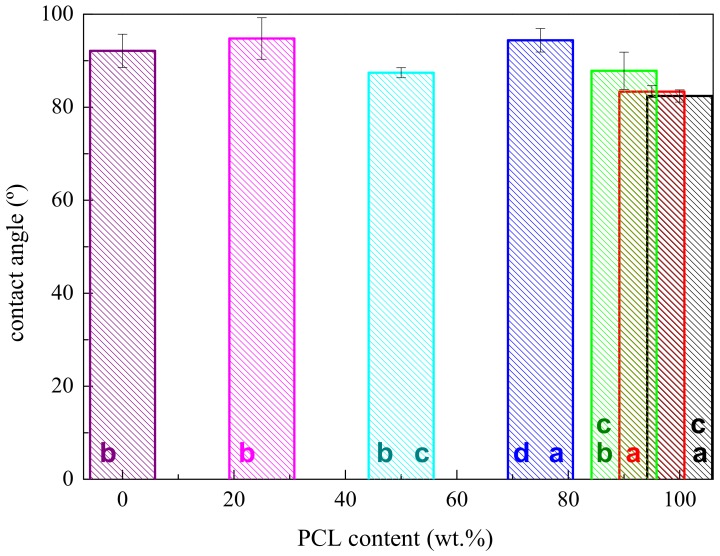
Dependence of contact angles values on PCL content in the blends. *Different letters (**a, b, c** and **d**) in the bars indicate statistically significant ANOVA differences between compositions (*p* < 0.05) applying multi-comparisons by Tukey post-hoc tests.

Both components are hydrophobic, but a higher hydrophobicity is exhibited by HDPE, as deduced from the contact angle values represented in Figure 2. This immiscibility gives rise to the formation of HDPE drops because it is at a smaller ratio in those specimens (samples PCL95, PCL90, and PCL75). In spite of the fact that segregation of these two polymers does undoubtedly exist, the lack of these blends, with the lowest HDPE content of extensive debonding at the interphase and non-existence of voids, seems to imply some compatibility between the two polymers. As will be commented later, this is corroborated by some other results, like those attained by FTIR, their crystallization behavior, and mechanical parameters.

A columnar-matrix morphology appears to be developed for the PCL50 blend (Figure 1e). This picture suggests very poor adhesion at the interface between the two components and, therefore, detrimental macroscopic mechanical properties are expected for this composition, as will be discussed later.

The shape of the domains completely changes in sample PCL25, when the PCL is the minor component. The droplets become ellipsoid-like, as observed in Figure 1f. This feature might be related to differences in hydrophobicity between PCL and HDPE, or to variations in their corresponding melt viscosities. Figure 2 shows that the former are not too large within the whole set of samples. In fact, the one-way ANOVA (*F_cri_*_t_ = 2.30, *p* = 0.05) results of the values for contact angles indicate that there are statistically different between group means (*F* = 18.6). The variations applying multi-comparisons of these mean values by Tukey post-hoc tests displays, through labeling with letters within the bars, show that not all of data are statistically different. This feature could be expected because, as aforementioned, both PCL and HDPE are hydrophobic.

Other very important variables affecting the morphological characteristics, include changes in the melt viscosities, since the processing temperature is rather high compared with PCL melting temperature. Further, at this composition, the PCL dispersed phase is fixed by the HDPE matrix during cooling because of the higher solidification temperature in the major component [40]. 

**Figure 3:**
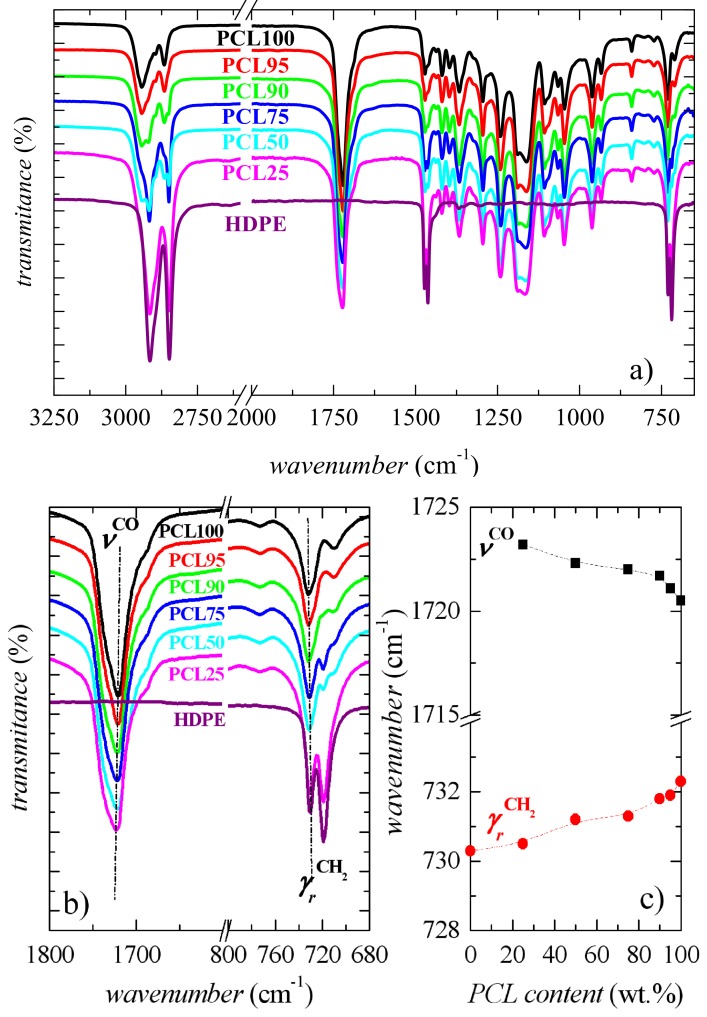
**a**) Overall Fourier Transform (FTIR) spectra; **b**) spectra at two wavenumber regions; and, **c**) dependence on PCL composition of the bands at about 1720 and 732 cm^−1^ for the different blends and the two homopolymers.

As aforementioned, the absence of voids at the interphase seems to indicate the existence of certain compatibility. It could be feasible, taken into account the relatively similar chemical structure between these two polymers, with the exception of ester groups in the architecture of PCL chains. FTIR experiments have been performed, in order to understand the interactions between these two components, 

The FTIR spectrum at room temperature of semicrystalline PCL exhibits the absorption bands, that are characteristic of a polyester composed of aliphatic linear chains (see Figure 3a). Thus, a doublet arising from the C−H stretching of the methylene groups is observed between around 2800 and 3000 cm^−1^, plus a band appearing at 1720–1725 cm^−1^, which is attributed to the carbonyl group. Additional bands appear ranging 700–1600 cm^−1^, which are associated with different modes of the polymer skeleton: Rocking, wagging, bending, and stretching of methylenes and also with *trans* and *gauche* isomerizations of ester groups, similarly to the case of PET [41]. On the other hand, characteristic peaks for HDPE, shown in Figure 3a are [42]: the one assigned to C−H stretching in the methylene groups at about 2850 and 2912 cm^−1^; the band associated with the C−H bending mode of methylene groups at 1470 cm^−1^; and the two peaks at around 730 and 720 cm^−1^ related to the CH_2_ rocking modes of the sequence of the CH_2_ groups in its paraffin structure.

Figure 3b represents two amplified spectral regions: The characteristic one for the carbonyl group, which is exclusively related to the PCL component, at about 1720 cm^−1^, and the zone associated with the rocking deformation modes that involve the CH_2_ groups from either, PCL or HDPE. The former exhibits a great intensity for PCL and for the different blends, independently of their composition, and as expected, it is not present in HDPE. The 1720 cm^−1^ band shows an asymmetric shape because it is actually composed of two constituents attributed to the crystalline and amorphous components of PCL [43]. In fact, the amorphous carbonyl band is located at 1735 cm^−1^ in the molten PCL state above 70 °C. On cooling, crystallization takes place and a narrower band with a maximum of around 1720 cm^−1^ is developed. These changes have been described as reversible on heating [43] and if the overlapped carbonyl bands can be resolved, their absorbances will provide an estimate of the PCL crystallinity.

Incorporation of HDPE moves the location of this asymmetric band to a slightly larger wavenumber, as clearly depicted in Figure 3c. This fact indicates that polyethylene chains can affect the amorphous or the crystalline PCL phases because some interactions can be established at interfaces. The existence of those local contacts between these two components is also deduced from the common region related to the rocking modes from the CH_2_ groups existing in both macromolecular chains. These results show that the phase segregation, observed from SEM pictures, does not occur in a complete extent and some interactions within the amorphous PCL and HDPE regions take place.

### 3.2. Crystalline Characteristics and Phase Transitions

**Figure 4:**
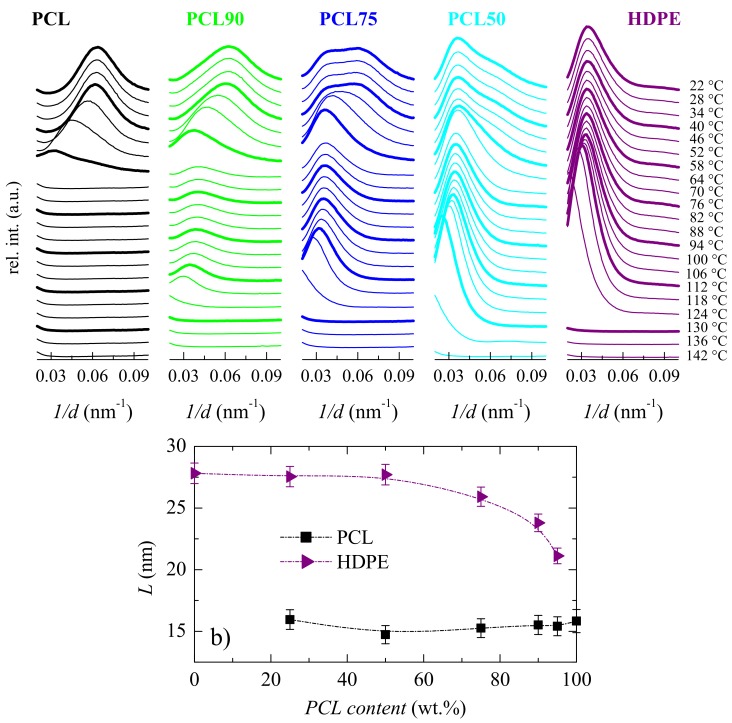
**a**) Real-time variable-temperature Lorentz-corrected SAXS profiles obtained with synchrotron radiation in a melting experiment from 20 to 144 °C at 8 °C/min for the indicated specimens. Only one every three frames is plotted for clarity; **b**) variation upon PCL composition of the long spacing values at room temperature for the PCL and HDPE crystallites.

Synchrotron measurements have been performed at either, small or wide angle to understand whether the existing interactions influence the crystalline regions in different blends. Figure 4 shows the SAXS results. The upper plot (Figure 4a) depicts that either, PCL or HDPE exhibits long spacing, i.e., both develop lamellar crystallites. At room temperature, the maximun of Lorentz-corrected SAXS profile for PCL appears at 0.063 nm^−1^, while for HDPE is observed at 0.036 nm^−1^, as deduced from Figure 4a. Lorentz-corrected patterns in the blends are clearly bimodal, being composed of the corresponding contribution from the two pristine homopolymers (at 0.063, and 0.036 nm^−1^, respectively). Their intensity is dependent on blend composition, as deduced from the plots for PCL90, PCL75, and PCL50, as examples. These SAXS profiles and their variation with blend composition (and temperature) indicate that both polymers develop their own crystals. The values of the most probable long spacing for the HDPE crystallites, represented in Figure 4b, are considerably much larger than the ones from PCL crystals. They are obtained as the inverse values from the maxima in the Lorentz-corrected patterns [44]. An important difference is observed at room temperature. Long spacing remains rather constant in PCL independently of its content in the blend, while it is significantly lowered for HDPE crystals at high PCL contents, as observed in Figure 4b. It follows that PCL composition is an important variable in the location of maxima and, consequently, value of long spacings.

**Figure 5:**
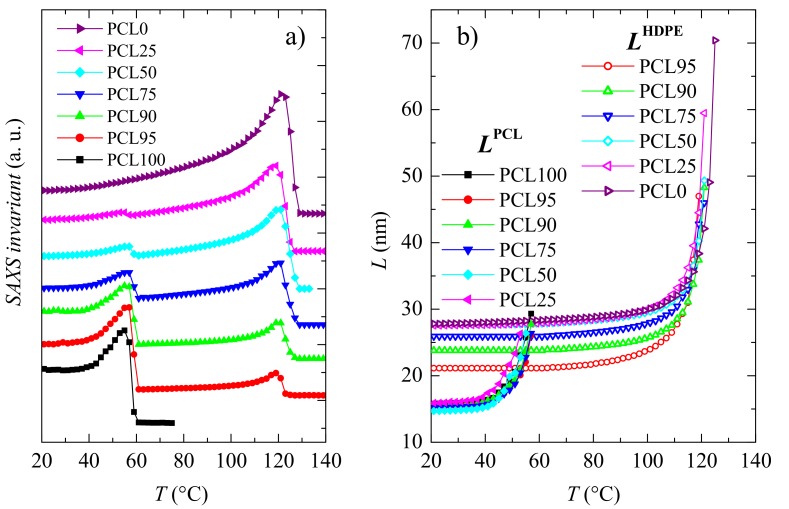
Temperature dependence, on melting, of: **a**) the SAXS relative invariant, and **b**) the most probable long spacings of the PCL and HDPE counterparts, corresponding to the neat homopolymers and their blends.

It is also noticeable in Figure 4a that the Lorentz-corrected peak from PCL disappears at quite low temperature because melting takes place. Consequently, an isotropic state is reached for PCL, while only the Lorentz-corrected maximum for HDPE remains in the blends. These features can be clearly deduced from the variation with temperature of two of the most important SAXS parameters: The relative invariant [44,45] and the long spacings. The former is shown in Figure 5a, where the melting of the two different crystal counterparts is well evident: The ones for PCL melting at around 60 °C and those for HDPE at around 120–125 °C. Obviously, the intensity of these two components shows a direct dependence with the blend composition.

The variation of these two long spacings on temperature is depicted in Figure 5b, where an important recrystallization process on heating is seen in all the samples and for the two types of crystals prior to their corresponding melting processes. This is accompanied by an important intensity increase, as observed from the invariant values represented in Figure 5a. Accordingly, their peak position is moved to lower values of 1/d and the intensity rises as temperature is raised. Besides, the long spacings of HDPE crystallites are considerably higher than those for PCL. Although, as mentioned above (see Figure 4b), for the values at room temperature, the long spacings of HDPE decrease as PCL content in the blend increases.

**Figure 6:**
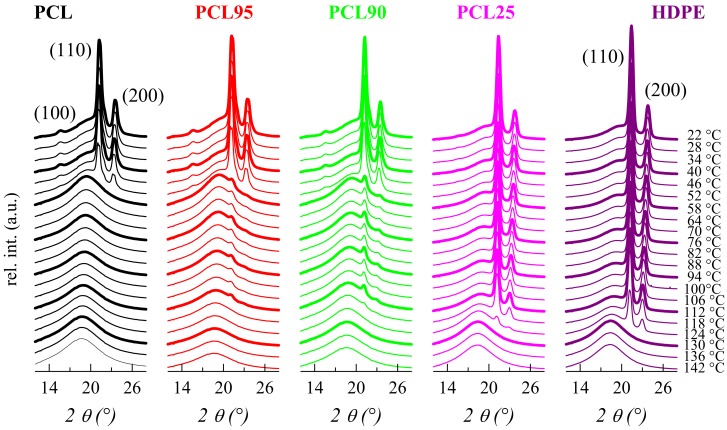
Real-time variable-temperature WAXS profiles obtained with synchrotron radiation for the indicated samples in a melting experiment at 8 °C/min. Only one every three frames is plotted for clarity.

Some more information is achieved from the WAXS patterns, since both PCL and HDPE are semicrystalline, as aforementioned. Both crystallize in their respective orthorhombic lattice but their profiles are very similar, in such a way that the WAXS profiles are much less informative than the previously commented SAXS ones. The (100) reflection in the PCL is the most distinguishing detail, as observed in Figure 6, but its intensity is too small to be sensible to changes in composition. The rest of the diffractions from either, PCL or HDPE appear at analogous positions. It is clearly evident that PCL melts at around 60 °C. HDPE melts at considerably higher temperature, independently of PCL content. These facts are in agreement with those attained from SAXS.

The overall degree of crystallinity, *f*_c_^WAXS^, at room temperature can be usually estimated from the WAXS diffractogram. In polyethylene-based materials, the amorphous component and the crystalline diffractions can be regularly deduced using a fitting program [46,47,48,49]. This allows the decomposition of the X-ray patterns and, consequently, the estimation of the resulting crystallinity. A more precise assessment requires knowledge of the actual amorphous component. An approach successfully used in polypropylene based derivatives [50,51,52] is attaining the amorphous molten profile from variable-temperature X-ray diffraction experiments, and determining the amorphous halo at a given temperature (room temperature, for instance) by considering the observed temperature coefficient. Once it is known, its subtraction from the actual crystalline profile allows the degree of crystallinity to be estimated. Unfortunately, the similarity found between the diffractograms either, at room temperature or in their molten state for these two polymers precludes the determination of their respective degrees of crystallinity when the two crystalline populations are present, as it happens at room temperature.

**Figure 7:**
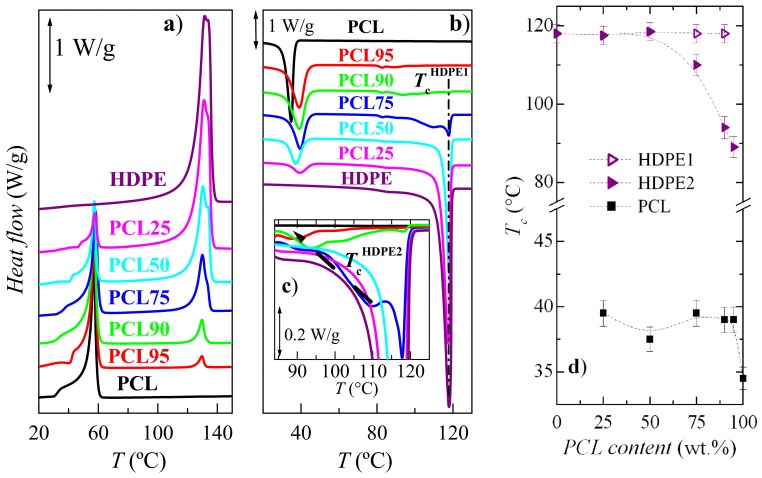
Differential scanning calorimetry (DSC) curves corresponding to; **a**) the first heating process, and **b**) the subsequent crystallization corresponding to the neat homopolymer and their blends; **c**) an enlargement in the HDPE crystallization region; and, **d**) variation upon PCL composition of the crystallization temperature (*T*_c_) for the crystallites of PCL and the two components of HDPE.

Figure 7 shows the DSC results obtained both for the first melting process and for the subsequent crystallization by cooling from the melt. The melting curves (Figure 7a) confirmed the fact that PCL melts at temperature much lower than HDPE. This difference seems to make HDPE a good candidate to enhance some PCL physical properties, as will be commented below.

Figure 7a is quite analogous to that derived from the dependence of SAXS invariant on temperature represented in Figure 5a. Thus, the PCL orthorhombic crystals melt at around 60 °C, as listed in Table 1, and those also orthorhombic belonging to the ordering within the HDPE chains reach the amorphous state at about 132 °C, rather independently of PCL composition in the blend. The important implication is that, in a certain temperature interval, the remaining HDPE ordered entities could provide rigidity to the already molten PCL chains.

Figure 7 shows a very complex melting process for the PCL constituent in the pristine polymer and all the blends. Thus, distinct endothermic events are noticeable, which are ascribed to the existence of several melting-recrystallization stages. Furthermore, its main melting peak is rather similar for all these samples, PCL and its blends, with the obvious exception of their intensity. This implies that, in the PCL-based materials, a development of the crystalline PCL structure occurs at ambient conditions, owing to the low melting and glass transition temperature [36] of PCL. It makes it mandatory to be very careful for obtaining consistent and reproducible results. Nevertheless, in the current samples, the melting temperature at the maximum for the PCL crystallites does not change much with PCL composition, as deduced from Table 1. This corroborates the constancy noticed in long spacing in these crystals at room temperature (see Figure 4b).

The results of the subsequent cooling process (Figure 7b) were very interesting as two opposite behaviors are deduced. On one hand, HDPE crystallization was significantly hindered by the presence of molten amorphous PCL macro-chains (see Figure 7b,c) as the minor component, pointing out again the existence of interactions between these two polymers. Accordingly, the main crystallization process was decomposed into several stages, with a small amount yet crystallizing at 118 °C, in PCL75 and PCL90, and the rest being delayed and appearing in distinct steps at lower temperatures, as observed in Figure 7c. In fact, the variation of the two main components for the crystallization of the HDPE counterpart is plotted in Figure 7d. It is observed that these two components merge already in blend PCL50. On the other hand, the existing HDPE crystallites exert a noticeable nucleating effect in the PCL crystallization, and it was shifted to a significantly higher temperature in the blends (see Figure 7b,d). 

Similarities that appear between Figure 4b and Figure 7d demonstrate the significant influence of composition on the way crystallization takes place and on the resulting long spacing values obtained. Nevertheless, and in spite of PCL crystallites are developed in the blends at *T*_c_ higher than in the pristine polymer, because of the nucleating effect of HDPE crystals, their average long spacing reached are analogous.

The overall degree of crystallinity for both crystalline components can be determined from the DSC curves during the first melting process. The results obtained for crystallinity after normalizing the enthalpies by the actual PCL or HDPE amounts, at each blend, are reported in Table 1. The incorporation of HDPE leads to a slight increase in the PCL crystallinity. In contrast, HDPE crystallinity is much more affected by the presence of PCL so that a clear decrease is observed as the PCL content increases. As commented above, the HDPE crystallization is significantly hindered by the presence of PCL macro-chains when it is the minor component.

All these features that are deduced from the morphological and structural analyses point out the importance of the partial compatibility of both isotropic melts. Favorable interactions are promoted, leading to changes in the crystallization of both components and in the ultimate characteristics of their crystalline regions. These structural details might exert noticeable effect in the ultimate performance of the different materials, which can determine their final usage. Here, attention has been focused on the evaluation of gas transport behavior and of the mechanical response. Both features can turn out of high interest for further bio-applications.

### 3.3. Transport Properties

In relation to the former, the permeation process in polymers is usually expressed in terms of the solution-diffusion model and Fick’s laws [53]. If the permeant concentration is sufficiently low, then the parameters appearing in the different expressions are presumed to be independent of the concentration. Accordingly, the variation with time (s) of the pressure (Pa) in the downstream part for a pressure step experiment becomes [54],

(1) $$p_{L}=\frac{APp_{0}}{Ve}\left(t-\frac{e^{2}}{6D}+\frac{2e^{2}}{\pi^{2}D}\sum_{n=1}^{\infty }\frac{{\left(-1\right)}^{n+1}}{n^{2}}\exp\left(-\frac{Dn^{2}\pi^{2}t}{e^{2}}\right)\right)$$

where *e* (m) represents the membrane thickness, *A* (m^2^) is the effective area of the film, *V* (m^3^) is the volume of the low-pressure chamber, *P* (mol · m/m^2^ · s· Pa) reads for the permeability, and *D* (m^2^/s) is the diffusion coefficient. The *p*_0_ and *p*_L_ represent pressures in the high-, and low-pressure sides, respectively (*p_H_* = *p_0_* when time *t* > 0), as specified in the Experimental Part.

The two parameters specific of the material are correlated by the expression,

*P* = *S* · *D* (2)

where *S* (mol/m^3^ · Pa) is the solubility coefficient. 

The asymptotic behavior of *p*_L_, deduced from steady-state measurements performed at ambient temperature, becomes linear for the various gases. Such asymptotic limit can be expressed as follows:

(3) $$\underset{t\to \infty }{\lim}\;p_{L}=\frac{APp_{0}t_{lag}}{Ve}\left(\frac{t}{t_{lag}}-1\right).$$

**Figure 8:**
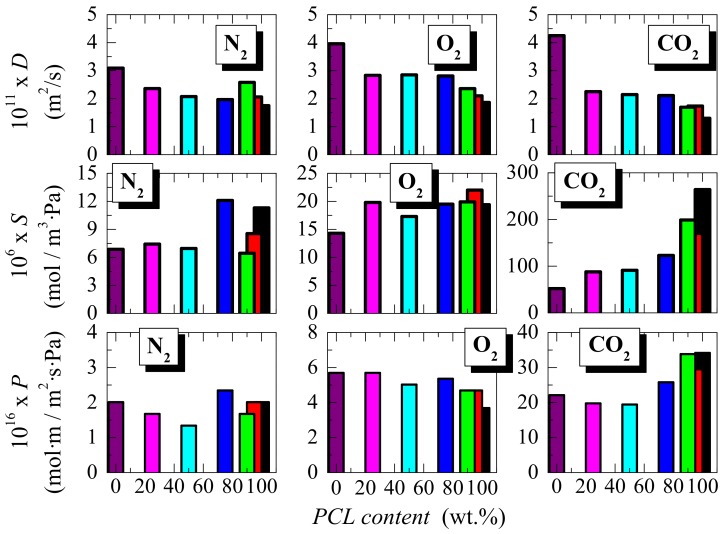
Dependence on PCL content of the diffusion, solubility, and permeability coefficients for N_2_, O_2_, and CO_2_ gases in tests performed at 30 °C, corresponding to the neat homopolymers and their blends.

The extrapolation to the abscissa axis (time) of this linear behavior defines the time lag as: *t*_lag_ = *e*^2^ / 6*D*, from where the diffusion coefficient, *D*, can be obtained for the different samples. The variation of *D* on PCL composition is depicted in the upper part of Figure 8. The value of *D* depends on the rate of the permeation development to attain steady-state conditions, and it is also related to the permeant size, the mobility, and structure of the matrix where the diffusion takes place [55]. In the particular case of semicrystalline polyolefins, this diffusion coefficient is reported by Michaels and Parker [56] to be given by,

(4) $$D=D^{*}/\tau \beta$$

being *D*^∗^ the diffusion coefficient of a totally amorphous polymer. In the presence of crystallites, the pathway of the diffusion is dependent on the tortuosity of the system, reflected by parameter τ, while *β* is primarily dependent on the mobility restrictions in those amorphous regions, which are located near the crystals [57]. Tortuosity in these specimens under analysis is mainly ascribed to the hindrance caused by presence of the PCL and HDPE crystallites.

Figure 8 depicts that the PCL homopolymer shows diffusivity coefficients for the three gases tested lower than those exhibited by HDPE. Moreover, the values in the blends at the different PCL compositions range from those observed in the pristine homopolymers. The results for the *S* and *P* coefficients, found in the distinct blends at the three gases obtained from Equations (2) and (3), are also presented in Figure 8. In general there is a trend that at a given gas decrease as PCL content is lowered since the neat HDPE exhibits a solubility coefficient inferior to that presented by PCL. This difference is much larger for the CO_2_ gas by a factor of around 5. This feature is ascribed to the mutual affinity between CO_2_ and PCL film as the PCL component is raised in the blend. However, permeability does not show a unique tendency and it strongly depends on the gas tested. Under N_2_, behavior goes through a minimum-maximum, a reduction that is noticed for O_2_, while a significant increase is attained for CO_2_, as the PCL content rises. All these features seem to indicate that variations in crystallinity, and in values of long spacing with composition, do not significantly affect the transport properties in these blends. This is also due to the fact that gases are primarily transported through amorphous regions. Moreover, the transport magnitudes, shown by the pristine PCL and HDPE polymers, are relatively analogous and the effect of structural changes is minimized. As commented in the Introduction, long-term drug delivery devices are one of the feasible bio-applications for these materials. It is important, then, to maintain or reduce permeability to keep the drugs intact over time. The characteristics found indicate that the effect of a small amount in HDPE does not significantly alter the gas transport properties of the PCL.

### 3.4. Mechanical and Viscoelastic Responses

**Figure 9:**
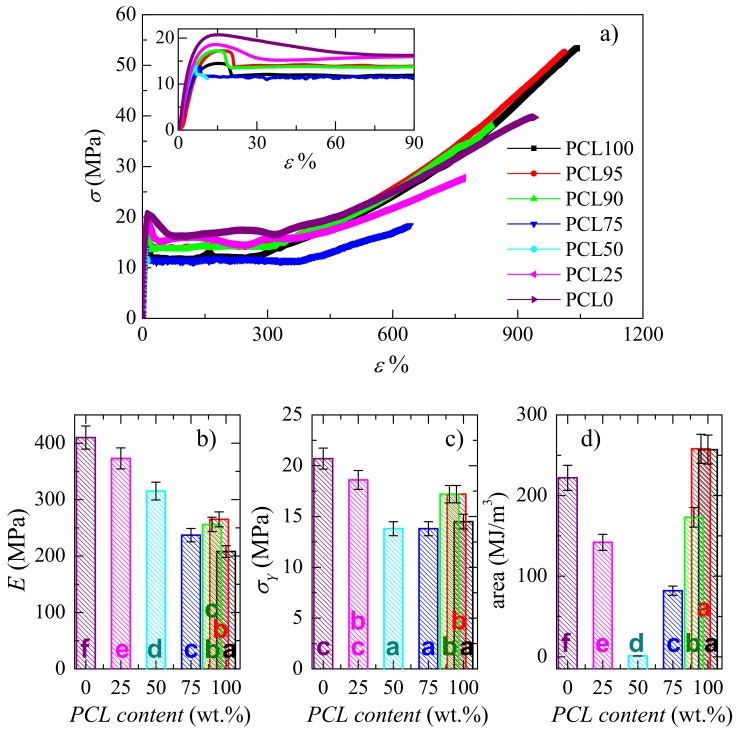
**a**) Stress-strain curves (at room temperature and at a rate of 10 mm/min) for the pristine PCL and HDPE homopolymers and the several blends. The inset enlarges the deformation X axis to better notice the differences; **b**), **c**) and **d**) dependence on PCL content of different mechanical parameters: Young´s modulus, stress at yield and area under the stress-strain curve. *Different letters in the bars for **b**), **c**) and **d**) plots indicate statistically significant ANOVA differences between compositions (*p* < 0.05) at each mechanical parameter applying multi-comparisons by Tukey post-hoc tests.

Regarding the mechanical behavior, Figure 9a shows the stress-strain curves and some mechanical parameters estimated: Young´s modulus (*E*), stress at yield (*σ*_Y_) and the area under curve, related to toughness, are represented in Figure 9b–d. The behavior deduced from these curves is typical of ductile polymer samples, except the one for PCL50 blend that shows poor adhesion at the interface between the two components, as aforementioned. Thus, the engineering deformation curves, shown in Figure 9a, exhibit three evident regions: An initial one, where the stress increases linearly with the strain, allowing the estimation of *E*; following this first stage, a yield region appears, characterized by a maximum in *σ*_Y_,, which can be observed in more detail in the inset of Figure 9a; and, finally, a third region where there is again an increase of the stress (between strain values from around 300 to 400%) so that a considerable strain-hardening is observed, related with stress-induced orientation of the polymer chains. Thus, the stress-strain curves of these blends are characterized by formation of a neck, as also deduced visually during the stretching progression. Another important aspect is that the yield region in PCL is narrower than in HDPE, what can be ascribed to the presence of two yield points in HDPE, as it has been described for other polyethylenes [58,59].

Moreover, the stress-strain measurements in these blends appeared to be reproducible, both in the shape of the stress-strain curves and also in the mechanical parameters deduced from the curves, which have been obtained for the various specimens stretched for a given sample (see Figure 9b–d). On the other hand, one-way ANOVA (*F*_crit_ = 2.57, *p* = 0.05) proves that there are statistically significant differences between group means of these reported mechanical properties (the *F* values for *E*, *σ*_Y_ and area below the stress-strain curve data are 27.4, 82.9, and 176.5, respectively). These statistical differences are also noticed when multi-comparisons are applied to all of these mean values by Tukey post-hoc tests. They are shown through labeling with letters within the bars in Figure 9b–d. Yield stress parameter is that exhibiting the less statistically significant differences.

Incorporating small HDPE amounts lead to stiffer materials and, consequently, the modulus value increases. The *E* variation is quite linear upon PCL composition with the exception of PLC95 and PCL90 blends, where a positive deviation of the mixing law seems to occur at these lowest HDPE contents, as depicted in Figure 9b. In fact, their *E* values are 265 and 256 MPa for PCL95, and PCL90, respectively, compared with 209 MPa in the pristine PCL, in spite of the significant reduction in these two blends of the HDPE crystallinity, their long spacing, and the size of their crystals, as reported in Table 1 and seen in Figure 5b. In addition, this enlargement in *E* value is observed in these two blends although phase separation exists between the two components, as shown in Figure 1b,c. This indicates that, not only is this mechanical parameter maintained, but even improved above 20%. An increase in rigidity is usually associated with a rise either, in the crystallinity or in the content of a hard component [48,49,60,61]. This positive deviation is also observed in the yield stress, *σ*_Y_, values at those lowest HDPE contents, as depicted in Figure 9c. The favorable variation of *E* was not shown in other blends based on PCL [14].

At the opposite interval of compositions, the comparison of Young´s modulus for PLC0 (HDPE) and PCL25 also shows a slightly positive deviation of mixing law in the PCL25 blend, as deduced from Figure 9b. Thus, the values achieved are 410, and 373 MPa, respectively. A slight improvement for this magnitude occurs in spite of the crystallites corresponding to the PCL component in PCL25 are much smaller than those from the HDPE, as can be deduced from Figure 5b, and taking into account the similarity in the degree of crystallinity within both ordered regions [44]. Accordingly, their reinforcement role is smaller. This feature seems to point out favorable interactions in the amorphous zones when HDPE is the major constituent. The value of yield stress for PCL25 is predicted by the mixing law (see Figure 9c). 

Tensile strength and toughness are also rather similar in PCL95 and PCL100. The former is 52.5 MPa for PCL95 and 53.5 MPa for the neat PCL100. Concerning the toughness of the blends, as measured by the area below the stress-strain curves and represented in Figure 9d, displays first that the toughness of PCL95 is very close to that exhibited by PCL100 and, secondly, that HDPE impairs the toughness of these blends at the rest of compositions. A minimum is shown in the PCL50 sample, which can be explained in terms of its morphology (see Figure 1d) and the subsequent lack of adhesion between both polymeric constituents. At this point, it should be commented that tensile strength and toughness are mechanical parameters, estimated from the non-linear regime, and their importance from a practical standpoint is not as significant as the modulus or the yield stress, except in polymers with very brittle mechanical behavior. This is not the case for PCL and HDPE, as they are ductile polymeric materials, and because of their blends, with the exception of PCL50. Thus, maintenance or the observed reduction is considered a good enough trend.

All these results indicate that the establishment of favorable interactions within the amorphous regions should be endorsed in blends of semicrystalline components as a feasible strategy to improve their global mechanical behavior. The effects are expected to be more relevant at compositions where the component crystallizing at higher temperature is a minority, as observed in the blends with low HDPE incorporations. 

**Figure 10:**
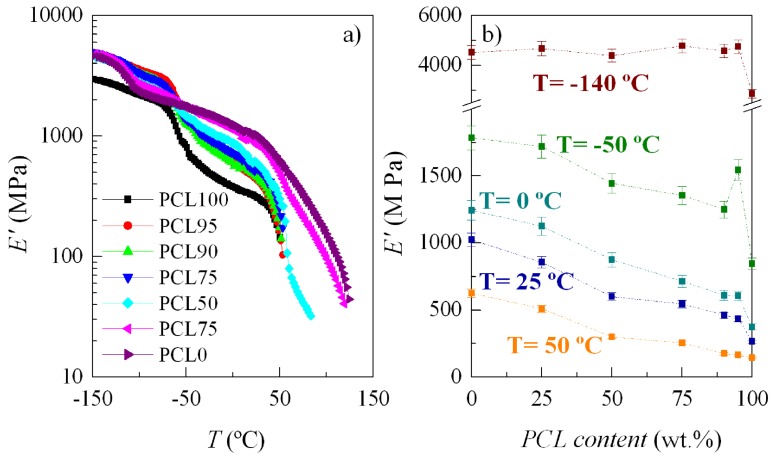
**a**) Dependence of storage modulus on temperature (at 3 Hz); and **b**) its variation upon PCL composition at distinct temperatures.

In order to confirm the positive deviation in rigidity, observed for those compositions, dynamic mechanical analysis has been also performed as a function of temperature. In addition, the relaxation processes have been examined. Figure 10a shows storage modulus, *E*′, dependence on temperature, while Figure 10b shows its variation at distinct temperatures upon PCL composition. It is noticeable that *E*′ values for the PCL95, PCL90, PCL75, PCL50, and PCL25 blends are in between of those corresponding to PCL and HDPE in the whole interval of temperature, excepted at very low temperatures below the γ relaxation of HDPE. The improvement in rigidity by incorporation of HDPE is clearly noticeable, even at the lowest HDPE content in the PCL95 blend. The positive deviation from the mixing law is again clearly observed, this being more important as temperature is lowered. At the highest temperature analyzed in Figure 10b, 50 °C, the relationship on PCL content becomes almost linear because a considerable part of PCL is in a molten state.

**Figure 11:**
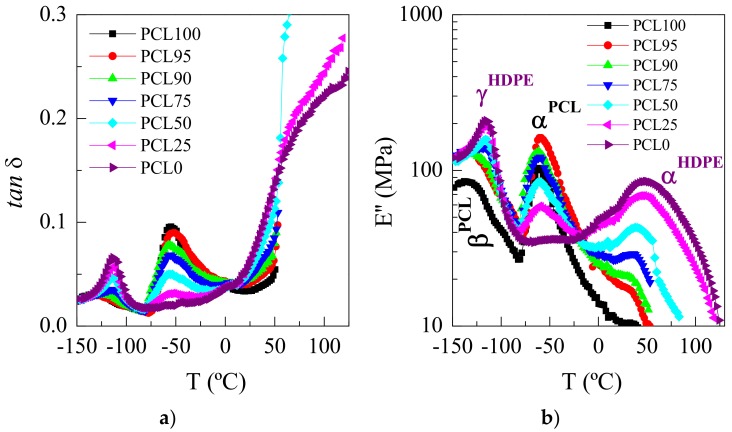
Temperature dependence of the imaginary magnitudes for the different samples: **a**) tan δ and, **b**) E″ representations, at 3 Hz.

Figure 11 shows the loss plots, either the tan *δ* or *E*″ representations, for the several specimens. Different relaxation mechanisms are observed: β^PCL^, γ^HDPE^, α^PCL^ and α^HDPE^ in order of increasing temperatures. As clearly noticeable, their location and intensity are dependent on PCL composition, similar to what has been described previously in other copolymers, blends, and composites [62,63,64,65].

The γ^HDPE^ relaxation in polyethylene was initially attributed to crankshaft motions in the polymethylenic chains [66]. However, the specific details for the motional process have not been completely ascertained. There is consensus about the fact that three or more CH_2_ units are involved in this relaxation [62]. The β^PCL^ relaxation is also related to local mobility with similar molecular origin [67]. Although, it takes place at lower temperature than in HDPE, probably because the lower values of its long spacing and, consequently, the presence of thinner crystals that imposed less restrictions in mobility to these amorphous regions.

The α^PCL^ process is attributed to generalized movements, involving long segments of polymeric chains, located in the amorphous regions of PCL, and this relaxation takes place during the corresponding glass transition of PCL [68,69]. The large decrease observed in the *E*′ values (Figure 10a) in the region of temperatures for those motions is explained by the cooperative character of this relaxation. Additionally, the maximum value of the tan *δ* intensity shows also a great decrease when the PCL content of the blends diminishes, considering the relative reduction of the amorphous content where this movement takes place, since the PCL amount is lowered. Moreover, a clear shift to slightly higher temperatures is observed in the location of the α^PCL^ relaxation in the PCL95 blend, fact that indicates a mobility restriction at this composition due probably to the existence of positive interactions at interfaces. The rest of the samples positions remain rather constant. 

Finally, the relaxation labeled as α^HDPE^, appearing at the highest temperatures in the *E*″ plots, is ascribed to polyethylene, and it is attributed to re-orientational and vibrational movements within the HDPE crystals [38,70]. The high-temperature side of this relaxation overlaps with the melting of those crystals. Moreover, the intensity of this relaxation decreases and a shift to lower temperature is observed as HDPE content decreases because of the smallest amount in HDPE crystals, and its thinner size, respectively.

## 4. Conclusions

Blends of poly(ε-caprolactone) and high-density polyethylene HDPE have been prepared at different compositions. The separation of both components is observed in the whole composition range, but the lack of an extensive debonding at interfaces, and the non-existence of voids, constitute the first evidence of some compatibility between these two polymers. This is further confirmed mainly by FTIR analysis and the properties evaluation.

In addition, both polymers are able to develop their own characteristic orthorhombic crystalline lattices. On heating, these two crystallites undergo melting-recrystallization processes in the different blends. The melting takes place in PCL, at temperature much lower than in HDPE, in all the blends, as confirmed by SAXS and DSC experiments.

When HDPE is the minor component, its crystallization is significantly hampered by the presence of molten amorphous PCL macrochains that point out again the existence of interactions between these two polymers. Those HDPE crystallites, generated at high temperature, exert a noticeable nucleating effect in the PCL crystallization and a shift to a higher temperature is observed in the blends at all the compositions. 

The addition of a small HDPE amount does not significantly alter the values of diffusivity and permeability coefficients of PCL gas transport. The solubility to CO_2_ changes considerably in the presence of HDPE, and an important decrease is observed as its content is increased in the blends.

The mechanical behavior displays an excellent balance in the PCL95 blend, compared with that exhibited by the neat PCL. The parameters related to rigidity increase, which shows a positive deviation of the mixing law, and the tensile strength and toughness are maintained. Moreover, the location of the α^PCL^ relaxation, which is related to the cooperative movements that occur along glass transition, shifts to a slightly higher temperature. All these features found at low HDPE contents confirm the existence of favorable interactions in the amorphous regions of both components. Consequently, if the biodegradability of PCL is not impaired by adding a small amount of HDPE (around 5 to 10% by weight), HDPE incorporation seems to be an appropriate strategy, and of low cost, for bio-applications of PCL requiring a higher mechanical performance.

## Figures and Tables

**Table 1:** Degree of crystallinity (normalized to the composition in the blend) and melting temperatures for the poly(ε-caprolactone) (PCL) and high-density polyethylene (HDPE) crystalline regions, determined from the first melting process for the different samples analyzed.

Specimen	*f_c_* ^PCL^	*T_m_*^PCL^ (°C)	*f_c_* ^HDPE^	*T_m_*^HDPE^ (°C)
PCL100	0.54	57.0	−	−
PCL95	0.54	57.5	0.36	129.5
PCL90	0.55	57.5	0.39	129.5
PCL75	0.56	58.0	0.51	130.0
PCL50	0.57	57.5	0.55	130.5
PCL25	0.57	57.5	0.60	131.5
PCL0	−	−	0.59	131.5

## References

[1] Van Natta F.J., Hill J.W., Carothers W.H. (1934). Polymerization and ring formation, ε-caprolactone and its polymers. J. Am. Chem. Soc..

[2] Huang S., Mark F., Bikales N., Overberger C., Menges G., Kroshwitz J. (1985). Biodegradable polymers. Encyclopedia of Polymer Science and Engineering.

[3] Kweona H.Y., Yoo M.K., Park I.K., Kim T.H., Lee H.C., Lee H.-S., Oh J.-S., Akaike T., Cho C.-S. (2003). A novel degradable polycaprolactone networks for tissue engineering. Biomaterials.

[4] Zein I., Hutmacher D.W., Tan K.C., Teoh S.H. (2002). Fused deposition modelling of novel scaffold architectures for tissue engineering applications. Biomaterials.

[5] Lee K.H., Kim H.Y., Khil M.S., Ra Y.M., Lee D.R. (2003). Characterization of nano-structured poly(epsilon-caprolactone) nonwoven mats via electrospinning. Polymer.

[6] Huang H., Oizumi S., Kojima N., Niino T., Sakai Y. (2007). Avidin–biotin binding-based cell seeding and perfusion culture of liver-derived cells in a porous scaffold with a three-dimensional interconnected flow-channel network. Biomaterials.

[7] Marrazzo C., Di Maio E., Iannace S. (2008). Conventional and nanometric nucleating agents in poly(epsilon-caprolactone) foaming: Crystals vs. bubbles nucleation. Polym. Eng. Sci..

[8] Luciani A., Coccoli V., Orsi S., Ambrosio L., Netti P.A. (2008). PCL microspheres based functional scaffolds by bottom-up approach with predefined microstructural properties and release profiles. Biomaterials.

[9] Sun H., Mei L., Song C., Cui X., Wang P. (2006). The in vivo degradation, absorption and excretion of PCL-based implant. Biomaterials.

[10] Dhanaraju M.D., Gopinath D., Ahmed M.R., Jayakumar R., Vamsadhara C. (2006). Characterization of polymeric poly(epsilon-caprolactone) injectable implant delivery system for the controlled delivery of contraceptive steroids. J. Biomed. Mater. Res. Part A.

[11] Manoukian O.S., Arul M.R., Sardashti N., Stedman T., James R., Rudraiah S., Kumbar S.G. (2018). Biodegradable polymeric injectable implants for long-term delivery of contraceptive drugs. J. Appl. Polym. Sci..

[12] Lim J.S., Ki C.S., Kim J.W., Lee K.G., Kang S.W., Kweon H.Y., Park Y.H. (2012). Fabrication and Evaluation of Poly(epsilon-caprolactone)/Silk Fibroin Blend Nanofibrous Scaffold. Biopolymers.

[13] Simoes M.C.R., Cragg S.M., Barbu E., De Sousa F.B. (2019). The potential of electrospun poly(methyl methacrylate)/polycaprolactone core-sheath fibers for drug delivery applications. J. Mater. Sci..

[14] Rosa D.S., Guedes C.G.F., Bardi M.A.G. (2007). Evaluation of thermal, mechanical and morphological properties of PCL/CA and PCL/CA/PE-g-GMA blends. Polym. Test..

[15] Arcana M., Bundjali B., Yudistira I., Jariah B., Sukria L. (2007). Study on properties of polymer blends from polypropylene with polycaprolactone and their biodegradability. Polym. J..

[16] Newman D., Laredo E., Bello A., Grillo A., Feijoo J.L., Müler A.J. (2009). Molecular Mobilities in Biodegradable Poly(DL-lactide)/Poly(ε-caprolactone) Blends. Macromolecules.

[17] Ravati S., Favis B.D. (2013). Interfacial coarsening of ternary polymer blends with partial and complete wetting structures. Polymer.

[18] Jing X., Mi H.Y., Huang H.X., Turng L.S. (2016). Shape memory thermoplastic polyurethane (TPU)/poly(epsilon-caprolactone) (PCL) blends as self-knotting sutures. J. Mech. Behav. Biomed. Mater..

[19] Seggiani M., Altieri R., Puccini M., Stefanelli E., Esposito A., Castellani F., Stanzione V., Vitolo S. (2018). Polycaprolactone-collagen hydrolysate thermoplastic blends: Processability and biodegradability/compostability. Polym. Degrad. Stab..

[20] Lee S.-H., Teramoto Y., Endo T. (2011). Cellulose nanofiber-reinforced polycaprolactone/polypropylene hybrid nanocomposite. Compos. Part A Appl. Sci. Manuf..

[21] Pan L., Pei X., He R., Wan Q., Wang J. (2012). Multiwall carbon nanotubes/polycaprolactone composites for bone tissue engineering application. Colloids Surf. B Biointerfaces.

[22] Boujemaoui A., Sanchez C.C., Engstrom J., Bruce C., Fogelstrom L., Carlmark A., Malmstrom E. (2017). Polycaprolactone Nanocomposites Reinforced with Cellulose Nanocrystals Surface-Modified via Covalent Grafting or Physisorption: A Comparative Study. ACS Appl. Mater. Interfaces.

[23] Mao L., Liu Y.J., Wu H.Q., Chen J.H., Yao J. (2017). Poly(epsilon-caprolactone) filled with polydopamine-coated high aspect ratio layered double hydroxide: Simultaneous enhancement of mechanical and barrier properties. Appl. Clay Sci..

[24] Lübbert C., Wendt K., Feisthammel J., Moter A., Lippmann N., Busch T., Mössner J., Hoffmeister A., Rodloff A.C. (2016). Epidemiology and Resistance Patterns of Bacterial and Fungal Colonization of Biliary Plastic Stents: A Prospective Cohort Study. PLoS ONE.

[25] Kwon C.I., Kim G., Jeong S., Lee D.H., Kim K.A., Ko K.H., Cho J.Y., Hong S.P. (2017). The Stent Patency and Migration Rate of Different Shaped Plastic Stents in Bile Flow Phantom Model and In Vivo Animal Bile Duct Dilation Model. Dig. Dis. Sci..

[26] Atrey A., Ward S.E., Khoshbin A., Hussain N., Bogoch E., Schemitsch E.H., Waddell J.P. (2017). Ten-year follow-up study of three alternative bearing surfaces used in total hip arthroplasty in young patients a prospective randomised controlled trial. Bone Jt. J..

[27] Kim J., Lew D.H., Roh T.S., Lee W.J. (2017). Use of Acellular Allogenic Dermal Matrix (MegaDerm) in Orbital Wall Reconstruction: A Comparison with Absorbable Mesh Plate and Porous Polyethylene. J. Craniofac. Surg..

[28] Tone S., Hasegawa M., Puppulin L., Pezzotti G., Sudo A. (2018). Surface modifications and oxidative degradation in MPC-grafted highly cross-linked polyethylene liners retrieved from short-term total hip arthroplasty. Acta Biomater..

[29] Saraiva J.A., da Fonseca T.S., da Silva G.F., Sasso-Cerri E., Guerreiro-Tanomaru J.M., Tanomaru M., Cerri P.S. (2018). Reduced interleukin-6 immunoexpression and birefringent collagen formation indicate that MTA Plus and MTA Fillapex are biocompatible. Biomed. Mater..

[30] Affatato S., Freccero N., Taddei P. (2016). The biomaterials challenge: A comparison of polyethylene wear using a hip joint simulator. J. Mech. Behav. Biomed. Mater..

[31] Kalfoglou N.K. (1983). Compatibility of Low-Density Polyethylene-Poly(ε-caprolactone) Blends. J. Appl. Polym. Sci..

[32] Moura I., Machado A.V., Duarte F.M., Nogueira R. (2011). Biodegradability Assessment of Aliphatic Polyesters-Based Blends Using Standard Methods. J. Appl. Polym. Sci..

[33] Chang H., Zhang J., Li L., Wang Z., Yang C., Takahashi I., Ozaki Y., Yan S. (2010). A Study on the Epitaxial Ordering Process of the Polycaprolactone on the Highly Oriented Polyethylene Substrate. Macromolecules.

[34] Cerrada M.L., Benavente R., Peña B., Pérez E. (2000). The effect of thermal treatment on the structure and relaxation processes of olefinic polymers synthesized with metallocene catalysts. Polymer.

[35] Nakagawa S., Kadena K., Ishizone T., Nojima S., Shimizu T., Yamaguchi K., Nakahama S. (2012). Crystallization Behavior and Crystal Orientation of Poly(ε-caprolactone) Homopolymers Confined in Nanocylinders: Effects of Nanocylinder Dimension. Macromolecules.

[36] Muñoz-Bonilla A., Cerrada M.L., Fernández-García M., Kubacka A., Ferrer M., Fernández-García M. (2013). Biodegradable Polycaprolactone-Titania Nanocomposites: Preparation, Characterization And Antimicrobial Properties. Int. J. Mol. Sci..

[37] Wunderlich B. (1980). Macromolecular Physics.

[38] Ward I.M. (1990). Mechanical Properties of Solid Polymers.

[39] Barton A.F.M. (1990). Handbook of Polymer-Liquid Interaction Parameters and Solubility Parameters.

[40] Zhang Y., Guo W., Zhang H., Wu C. (2009). Influence of chain extension on the compatibilization and properties of recycled poly(ethylene terephthalate)/linear low density polyethylene blends. Polym. Degrad. Stab..

[41] Chen Z., Hay J.N., Jenkins M.J. (2012). FTIR spectroscopic analysis of poly(ethylene terephthalate) on crystallization. Eur. Polym. J..

[42] Gulmine J.V., Janissek P.R., Heise H.M., Akcelrud L. (2002). Polyethylene characterization by FTIR. Polym. Test..

[43] Phillipson K., Hay J., Jenkins M. (2014). Thermal analysis FTIR spectroscopy of poly(ε-caprolactone). Thermochim. Acta.

[44] Ryan A.J., Stanford J.L., Bras W., Nye T.M.W. (1997). Biodegradable Polycaprolactone-Titania Nanocomposites: Preparation, Characterization and Antimicrobial Properties. Polymer.

[45] Baltá-Calleja F.J., Vonk C.G. (1989). X-ray Scattering of Synthetic Polymers.

[46] Cerrada M.L., Benavente R., Pérez E. (2001). Influence of thermal history on morphology and viscoelastic behavior of ethylene-1-octene copolymers synthesized with metallocene catalysts. J. Mater. Res..

[47] Cerrada M.L., Benavente R., Pérez E. (2002). Crystalline structure and viscoelastic behavior in composites of a metallocenic ethylene-1-octene copolymer and glass fiber. Macromol. Chem. Phys..

[48] Cerrada M.L., Pérez E., Lourenço J.P., Campos J.M., Ribeiro M.R. (2010). Hybrid HDPE/MCM-41 Nanocomposites: Crystalline Structure and Viscoelastic Behaviour. Microporous Microporous Mater..

[49] Cerrada M.L., Bento A., Pérez E., Lorenzo V., Lourenço J.P., Ribeiro M.R. (2016). Hybrid Materials Based on Polyethylene and MCM-41 Particles Functionalized with Silanes: Catalytic Aspects of In Situ Polymerization, Crystalline Features and Mechanical Properties. Microporous Microporous Mater..

[50] Pérez E., Cerrada M.L., Benavente R., Gómez-Elvira J.M. (2011). Enhancing the formation of the new trigonal polymorph in isotactic propene-1-pentene copolymers: Determination of the X-ray crystallinity. Macromol. Res..

[51] Pérez E., Gómez-Elvira J.M., Benavente R., Cerrada M.L. (2012). Tailoring the Formation Rate of the Mesophase in Random Propylene-co-1-Pentene Copolymers. Macromolecules.

[52] García-Peñas A., Gómez-Elvira J.M., Barranco-García R., Pérez E., Cerrada M.L. (2016). Trigonal form as a tool for tuning mechanical behavior in poly(propylene-co-1-pentene-co-1-heptene) terpolymers. Polymer.

[53] Wijmans J.G., Baker R.W. (1995). The solution-diffusion model: A review. J. Membr. Sci..

[54] Rutherford S.W., Do D.D. (1997). Review of time lag permeation technique as a method for characterisation of porous media and membranes. Adsorption.

[55] Michaels A.S., Bixler H.J. (1961). Flow of gases through polyethylene. J. Polym. Sci..

[56] Michaels A.S., Parker R.B. (1959). Sorption and flow of gases in polyethylene. J. Polym. Sci..

[57] Laguna M.F., Cerrada M.L., Benavente R., Pérez E. (2003). Effect of the comonomer content on the permeation behavior in polyolefin films synthesized with metallocene catalysts. J. Membr. Sci..

[58] Seguela R., Rietsch F. (1990). Double yield point in polyethylene under tensile loading. J. Mater. Sci. Lett..

[59] Brooks N.W., Duckett R.A., Ward I.M. (1992). Investigation into double yield points in polyethylene. Polymer.

[60] Fonseca C., Pereña J.M., Benavente R., Cerrada M.L., Bello A., Pérez E. (1995). Microhardness and thermal study of the annealing effects in vinyl alcohol-ethylene copolymers. Polymer.

[61] Cerrada M.L., de la Fuente J.L., Fernández-García M., Madruga E.L. (2001). Viscoelastic and mechanical properties of poly(butyl acrylate-g-styrene) copolymers. Polymer.

[62] Cerrada M.L., Pereña J.M., Benavente R., Pérez E. (2000). Viscoelastic processes in vinyl alcohol-ethylene copolymers. Influence of composition and thermal treatment. Polymer.

[63] de la Fuente J.L., Fernández-García M., Cerrada M.L. (2003). Viscoelastic behavior of a HTPB gum and its highly filled composites. Effect of the type of filler on the relaxation processes. J. Appl. Polym. Sci..

[64] Prieto O., Pereña J.M., Benavente R., Pérez E., Cerrada M.L. (2003). Viscoelastic relaxation mechanisms of conventional polypropylene toughened by a plastomer. J. Polym. Sci. Part B Polym. Phys..

[65] De la Fuente J.L., Wilhelm M., Spiess H.W., Madruga E.L., Fernández Garcia M., Cerrada M.L. (2005). Thermal, Morphological and Rheological Characterization of Poly(Acrylic Acid–*g*–Styrene) Amphiphilic Graft Copolymers. Polymer.

[66] Schatzki T.F. (1962). Statistical computation of distribution functions of dimensions of macromolecules. J. Polym. Sci..

[67] Sabater i Serra R., Escobar Ivirico J.L., Meseguer Dueñas J.M., Andrio Balado A., Gómez Ribelles J.L., Salmerón Sánchez M. (2007). Dielectric relaxation spectrum of poly (ε-caprolactone) networks hydrophilized by copolymerization with 2-hydroxyethyl acrylate. Eur. Phys. J. E.

[68] Harrison K.L., Jenkins M.J. (2004). The effect of crystallinity and water absorption on the dynamic mechanical relaxation behaviour of polycaprolactone. Polym. Int..

[69] Nikolic M.S., Mitric M., Dapcevic A., Djonlagic J. (2016). Viscoelastic properties of poly(ε-caprolactone)/clay nanocomposites in solid and in melt state. J. Appl. Polym. Sci..

[70] McCrum B., Read B., Williams G. (1991). Anelastic and Dielectric Effects in Polymeric Solids.

